# Improvement of Outcomes for Women With HIV Infection and Cervical Cancer

**DOI:** 10.1200/JCO.2016.69.0784

**Published:** 2016-08-29

**Authors:** Linda R. Mileshkin, Alison E. Freimund

**Affiliations:** Linda R. Mileshkin and Alison E. Freimund, Peter MacCallum Cancer Centre, Melbourne, Victoria, Australia

Patients with HIV infection are at an increased risk for certain cancers. With 36.7 million individuals estimated to be living with HIV worldwide, only 46% receive antiretroviral treatment.^1^ Human papillomavirus (HPV) –associated malignancies occur in excess among patients with HIV, with cervical cancer designated as an AIDS-defining condition.^2^ Of note, although the introduction of highly active antiretroviral treatment (HAART) has reduced the incidence of some cancer types in those living with HIV, such as Kaposi’s sarcoma and CNS non-Hodgkin lymphoma, the incidence of cervical cancer has not decreased. The link among HPV, HIV, and cervical cancer is becoming better understood and attributed to enhanced HPV carcinogenesis in the setting of HIV-related immunosuppression as well as more frequent infections, with multiple and/or high-risk HPV subtypes in women with HIV.^3^ In a study of > 309,000 US patients with HIV from 5 years before the date of HIV onset to 5 years after, the incidence of cervical cancer was documented to be significantly increased in women with HIV infection (relative risk, 5.4; 95% CI, 3.9 to 7.2).^4^

Some evidence also suggests that cervical cancer is more aggressive in women with HIV, who are more likely to present with more advanced-stage disease and respond less well to therapy. Cervical cancer remains the fourth leading cause of cancer death in women worldwide and is the leading cause of cancer death in women with HIV in sub-Saharan Africa.^5,6^ Because the majority of the disease burden is seen in low- and middle-income countries, with regions of a high prevalence of cervical cancer corresponding with regions of a high prevalence of HIV infection, our understanding of the impact of concurrent HIV on cervical cancer and its response to treatment is paramount.^7^

The implementation of routine Papanicolaou smears in developed countries has led to the detection of early cervical cancers curable with surgery alone. Similarly, the development of the cervical cancer vaccine is expected to significantly reduce the incidence of cervical cancer over the next 60 years.^8^ Despite this, women in the developing world and even disadvantaged areas of developed countries continue to have low vaccination and Papanicolaou test rates, which result in more locally advanced stages of cervical cancer at diagnosis.^9-12^

For example, in Botswana, a country in sub-Saharan Africa, cervical cancer is the leading cause of cancer death in women, with more than two thirds of cases occurring in women with HIV infection. The national prevalence of HIV in Botswana is 17% to 24%.^13^ In South Africa, cervical cancer is the most common cancer; however, despite this, the country’s cancer screening policy is only able to offer asymptomatic women three free cervical smears in a lifetime, which begin at age 30 years and are performed 10 years apart. For women with HIV and CD4 T-cell counts < 350 cells/μL, screening is done annually.^14^ The problem is compounded by the lack of knowledge about the survival interplay between HIV and cervical cancer. However, should these dual diagnoses predict worse outcomes, then a modified treatment approach may be required. Most concerning is that the last major improvement in cervical cancer survival was based on reports published 17 years ago and later confirmed in a large meta-analysis in 2008,^15-17^ which demonstrated the superiority of cisplatin-based chemoradiation to radiation therapy alone and resulted in a low 58% versus 50% 5-year disease-free survival. No subgroup analysis of the potential confounding effects of a concurrent HIV infection on outcome was possible because patients with HIV are commonly excluded from clinical trials in oncology.

In the article that accompanies this editorial, Dryden-Peterson et al^18^ present results of a prospective cohort of 348 women with cervical cancer treated in Botswana from 2010 to 2015. The primary objective was to determine the impact of concurrent HIV infection, which was present in two thirds of the cohort, on survival. Their conclusion is that despite good access to and use of antiretroviral treatment in 82% of the women before a cancer diagnosis, HIV infection significantly decreases survival from cervical cancer. Specifically, 3-year survival for the group with HIV infection was only 35% (95% CI, 27% to 44%) compared with 48% in the group without HIV infection (95% CI, 35% to 60%), with the majority of the deaths attributable to the cancer rather than to HIV. This occurred despite the fact that patients with HIV were younger, (median age, 42 years) compared with patients without HIV (median age, 58 years), had significantly greater access to education, and higher measures of wealth than those without HIV.

Another concerning finding is that although the majority (82.9%) of patients were considered candidates for potentially curative therapy, the radiotherapy completion rates were far from ideal. Specifically, 30.6% of the cohort were considered to have received an inadequate radiotherapy dose, with only 61.2% completing the planned brachytherapy, some of which had to be administered in another country. Furthermore, only 80.8% received at least one dose of concurrent cisplatin. However, these findings were not specifically different between the study groups and highlight the challenges of delivering a complex treatment like chemoradiation for cervical cancer in a developing nation and the need to better understand barriers to treatment completion in all women with cervical cancer. Of note, rates of radiation toxicity did not seem to be different between patients with and without HIV or necessarily the reason for the low rates of completion of optimal treatment, which the study by Dryden-Peterson et al^18^ was not able to explain fully. This finding is different from the small number of other studies in the literature reviewed in a recent Cochrane analysis, which suggested that toxicity from radiotherapy for cervical cancer generally is higher in patients with HIV.^19,20^ Unfortunately, no guidelines for the treatment of locally advanced cervical cancer in women with HIV have been published in the past decade. However, the Cochrane review found that patients who were started on HAART early had higher rates of treatment completion, and HAART has been recommended to be initiated as soon as possible in patients with HIV and newly diagnosed cervical cancer to reduce treatment toxicity.

Dryden-Peterson et al^18^ address important questions given the burden of both HIV and cervical cancer in less-developed countries and emerging initiatives to establish chemoradiation treatment in these countries. However, several methodological concerns limit the generalizability of the findings. The cohort included a mix of patients treated with palliative and curative intent; the median follow-up of 15.1 months is relatively short, with limited follow-up information available because the treating team did not follow-up with patients after treatment completion. Despite this, an analysis restricted to women who received curative intent therapy and guideline-concordant curative intent treatment was presented and suggests that HIV infection nearly doubled the risk of death.

Another concern is that the diagnostic work-up consisted of a clinical examination, chest x-ray, and abdominal ultrasound with no use of positron emission tomography scanning or magnetic resonance imaging. Hence, no information is available about tumor volume or nodal status, which are considered some of the most important factors in predicting prognosis in locally advanced cervical cancer. Without the inclusion of these baseline characteristics in stratification, imbalances between cohorts are likely to exist and potentially affect the validity and reproducibility of the findings.

In addition, Dryden-Peterson et al^18^ had limited information about the amount or number of cycles of concurrent cisplatin received during radiation, and although the study defined receipt of at least one cycle as guideline-concordant therapy, this is not consistent with the international standard recommendation to deliver cisplatin 40 mg/m^2^ once per week during pelvic radiotherapy. This issue is a significant confounder in the analysis given the established survival benefit from concurrent cisplatin therapy in the treatment of locally advanced disease.

Despite methodological concerns and the absence of detailed data about certain aspects, this study describes the importance of further research and investment into improving the outcomes of women with both HIV and cervical cancer. International collaboration through research networks, such as the recently established Cervix Cancer Research Network,^21^ that could provide infrastructure, quality assurance, financial support, data sharing, education, and expertise in conducting clinical trials can potentially help establish future initiatives to answer outstanding questions such as those highlighted by Dryden-Peterson et al.^18^ We commend these authors for their dedication to improve treatment for women with HIV and cervical cancer in this rising burden of disease that has had limited research to date.

One clear major barrier to optimal treatment of cervical cancer, and indeed many cancers, is access to radiation therapy. A recent analysis of radiation therapy infrastructure in 139 low- and middle-income countries found that only four (2.87%) have the requisite number of teletherapy units to manage the estimated burden of cancer in 2020 and that 55 (39.5%) have no radiation facilities.^22^ Another analysis of radiotherapy resources found that brachytherapy is available in only 20 of 52 African countries.^23^ If we are to reduce the number of deaths from cervical cancer in women with and without HIV, it is paramount that access to screening, vaccination, and treatment facilities in those areas of the world with the greatest burden of disease is addressed.
